# Oral Microbiota of Infants in Maternal Gestational Diabetes: A Systematic Review

**DOI:** 10.3390/children11040421

**Published:** 2024-04-02

**Authors:** Nicole Camoni, Giulio Conti, Alessandra Majorana, Elena Bardellini, Claudia Salerno, Thomas Gerard Wolf, Guglielmo Campus, Maria Grazia Cagetti

**Affiliations:** 1 ASST Valle Olona, Dental Unit, 21052 Gallarate, Italy; nicole.camoni@asst-valleolona.it; 2 Department of Biomedical, Surgical and Dental Sciences, University of Milan, 20112 Milano, Italy; 3 Department of Medicine and Surgery, School of Dentistry, University of Insubria, 21100 Varese, Italy; giulio.conti@uninsubria.it; 4 Department of Oral Medicine and Paediatric Dentistry, University of Brescia, 25121 Brescia, Italy; alessandra.majorana@unibs.it (A.M.); elena.bardellini@unibs.it (E.B.); 5 Department of Restorative, Preventive and Pediatric Dentistry, School of Dental Medicine, University of Bern, 3012 Bern, Switzerland; claudia.salerno@students.unibe.ch (C.S.); thomas.wolf@unibe.ch (T.G.W.); guglielmo.campus@unibe.ch (G.C.); 6 Graduate School for Health Sciences, University of Bern, 3012 Bern, Switzerland; 7 Department of Periodontology and Operative Dentistry, University Medical Center of the Jhoannes Gutenberg University Mainz, 55116 Mainz, Germany

**Keywords:** gestational diabetes mellitus, microbiota, oral microbiota, newborn microbiota

## Abstract

Gestational diabetes mellitus (GDM) affects approximately 5–20% of pregnant women and is associated with adverse pregnancy outcomes. This review aimed to assess whether the oral microbiota of infants and their mothers with GDM had a different composition from that found in unaffected women and offspring. PubMed, Embase, Scopus, and Google Scholar were searched in December 2023 after protocol registration in the International Prospective Register of Systematic Reviews (CRD42023406505). Risk of bias was assessed using the Joanna Briggs Institute Critical Appraisal tools. Overall, 1113 articles were identified; after evaluating the full texts, 12 papers were included in the qualitative analysis. In six studies of the eight included, significant differences in microbiota between M-GDM and M-nGDM were found. In four studies, a depletion of *Firmicutes* and an enrichment of *Proteobacteria* was found in the microbiota of infants. Since all included studies were judged to have high risk of bias, a quantitative synthesis of the results was not carried out. In conclusion, although the oral microbiota of infants from mothers with GDM could be different from that of infants from mothers without GDM, there is insufficient evidence to clarify this aspect so far.

## 1. Introduction

Human bodies developed eubiotic relationships with microorganisms harbored in various niches over time, reflecting evolutionary selection pressures [1,2]. When the balance between host and bacteria is present, the individual is generally healthy, while microbial imbalance favors the development of diseases [1,3]. Human bacteria not only interact with host by producing enzymes and protecting against external pathogens, but they also interact through their genome and coding activity. Although the terms microbiota and microbiome are used interchangeably, when referring to a microbial community, it is important to recognize the differences. The microbiome refers to the collective genomes of microorganisms, while the microbiota is the range of microorganisms present in the community [2,4]. It is generally recognized that the establishment of the microbiota in infants begins at the time of birth (postnatal colonization); this means that the initiation of microbiota colonization in infants is a dynamic process that starts during delivery and continues after birth [5,6]. The importance of the oral microbiota of infants has recently been emphasized. During the early stages of infant development, it is becoming crucial to recognize the interactions and adaptations of microbial communities to changing conditions that could result in alterations of the host environment and, consequently, play a role in the initiation and/or progression of diseases [7].

Pregnancy also seems to be a special time in women’s lives from a microbiological point of view, as hormonal changes play a role in modifying the microbiota [8]. If a disease occurs during gestation, the dysbiosis may be even more evident, as in the case of gestational diabetes mellitus (GDM) [9,10]. GDM is defined as any degree of glucose intolerance with onset or first recognition during pregnancy [11].

GDM is currently one of the most common complications of pregnancy, occurring in about 5–20% of women, and its prevalence is rising [12]. It is related with overweight and obesity, later age, a family history of type 2 diabetes mellitus, and ethnicity [13,14]. Diagnosis is normally achieved using the oral glucose tolerance test (OGTT), routinely performed during pregnancy [13]. The condition of prediabetes is also frequent and could be an adjunctive target of evaluation, even if there is still paucity of data [15,16]. Women positive for GDM are asked for dietary modification and increased physical activity, but insulin is used when glycemic control is not achieved [13]. The complications related with the infants of GDM mothers are stillbirth, congenital malformations, being large for gestational age, pre-term birth, birth trauma, hypoglycemia, hyper biliuria, and respiratory distress syndrome [17]. 

GDM affects the oral health of future mothers, especially when gingivitis or pre-conception periodontal disease is present [18]. These two diseases have a wide prevalence varying from 44.7 to 65.6% and from 17.93 to 35.20%, respectively [19]. The etiology of GDM was linked to oral inflammation due to higher levels of tumor necrosis factor, interleukin 6, and C-reactive protein [18,19]. Moreover, like GDM, periodontal disease is linked to miscarriages and pre-term delivery [20,21]. Women with GDM appear to be 6.43 times more likely to develop type 2 diabetes than non-GDM subjects [22]. Infants of mothers with GDM also appear to be at increased risk of developing type 2 diabetes in adulthood; the cumulative risk of being diagnosed with diabetes by age 20 is 15% and the prevalence of diabetes is 21% at age 22 [23,24]. Additionally, the inflammatory state due to GDM seems to influence the oral microbiota of mothers, in which dysbiosis appears to be present, as well as the oral microbiota of their infants, which seems to be different from that of children born to unaffected mothers [25]. The temporal variation in the composition and diversity of the microbiota and its association with different medical conditions, such as the peculiar characteristics of the oral microbiota of infants and mothers with GDM and what they share with periodontal patients, are still unclear; indeed, the long-term effects of crosstalk of the mother–infant microbiota need to be clarified [26].

Microbiota diversity can be assessed through indices that condense ecological data into a single value that takes into account both species richness (number of different species in a community) and uniformity (relative abundance of species) [27]. While an increase in the number of species or a more even distribution of their abundances results in a higher diversity score, indices differ in their sensitivity to these two components of richness and evenness. The discrepancies between common indices of community diversity, e.g., Shannon’s or Simpson’s index, have long been recognized in the field of ecology [28]. Given this premise, this systematic review was designed and conducted to gather scientific evidence on the diversity of the oral microbiota in infants and mothers with GDM compared to unaffected infants and mothers, considering microbiological data from culture, PCR, or sequencing.

## 2. Materials and Methods

### 2.1. Protocol and Registration

The review protocol was registered in the International Register of Systematic Reviews (PROSPERO) in March 2023 (CRD42023406505). The writing of this systematic review follows the Preferred Reporting Items for Systematic Reviews and Meta-Analyses (PRISMA) guidelines (Supplementary Materials File S1, PRISMA checklist).

### 2.2. Eligibility Criteria

A question was formulated according to PECO as follows: Do infants born to mothers with gestational diabetes as well as the mothers themselves have a different oral microbiota from that found in infants of unaffected mothers?

- P: infants born from mothers with GDM and women without GDM;
- E: oral microbiota analysis in saliva or mucosal or plaque samples;
- C: infants and pregnant women without any type of diabetic condition;
- O: oral microbiota dysbiosis/modification of composition, considering results from oral swabs and saliva/plaque samples detected by culturing, PCR, or DNA/RNA sequencing.

Clinical trials and interventional and observational studies were considered from January 2013 to December 2023. The exclusion criteria were studies including participants without any oral microbiota analysis for both infants and mothers. Furthermore, studies with infant samples older than 2 years and studies on women with serious health conditions other than diabetes, which could have altered the results, were excluded.

### 2.3. Search Strategy

A search was conducted using four databases: PubMed (National Library of Medicine), Embase (Elsevier), Scopus (Elsevier), and Google Scholar. The search was performed in March 2023 and updated in December 2023. The search strategy carried out for each database is displayed in the supplementary file (Supplementary Materials File S2, Electronic search). All references were uploaded to the Endnote 20^®^ software for duplicate management and study selection. Finally, the reference lists of the studies included were hand-searched to identify additional records.

### 2.4. Study Selection and Data Extraction

After duplicate exclusion, two independent authors (NC and GCo) screened the records by title and abstract; when in doubt, the opinion of a third author was requested (GCa). The selected papers were then screened in the full-text format by the same two authors. When a consensus was reached, the main characteristics of full texts were extracted and reported in a Microsoft Office Excel 2019^®^ spreadsheet. Data extraction was performed in duplicate by two authors (NC and MGC), including a description of the study design, outcome, variables evaluated, and results. Every effort was made to obtain original data from authors when needed; they were contacted via e-mail and/or ResearchGate^®^. Cohen’s kappa value for inter-reviewer agreement for both title/abstract and full text evaluation was performed.

### 2.5. Risk of Bias

The risk of bias was assessed using the Joanna Briggs Institute Critical Appraisal tools for cohort studies and cross-sectional studies [29]. The risk was considered low when all criteria were met or no more than 1 criterion was judged unclear; medium if 2 criteria were judged unclear and the others were met, or 1 criterion was not met and the others were met; or high if 3 or more criteria were judged unclear and the others were met, or 2 criteria were not met, and the others were met. Two reviewers (AM and EB) carried out the assessments and divergences were resolved with discussion.

### 2.6. Outcome Measures

The primary outcome for this review was the diversity in the composition of the oral microbiota of infants and pregnant women with GDM/prediabetes compared to that found in unaffected women and their offspring. The secondary outcome was the assessment of oral microbiota composition in relation to the type of delivery. 

### 2.7. Synthesis of the Results and Metanalysis

The Stata 18^®^ package was employed for the analysis. A meta-analysis was considered appropriate in the presence of studies with comparable data, i.e., reporting the same outcome and interventions, but it was not considered appropriate if the studies were jugged to be at high risk of bias as the result could be seriously misleading. In this condition, only a qualitative description of the results was possible. A *p*-value of 0.05 or less was considered statistically significant for all analyses.

## 3. Results

### 3.1. Study Selection 

**Figure 1:**
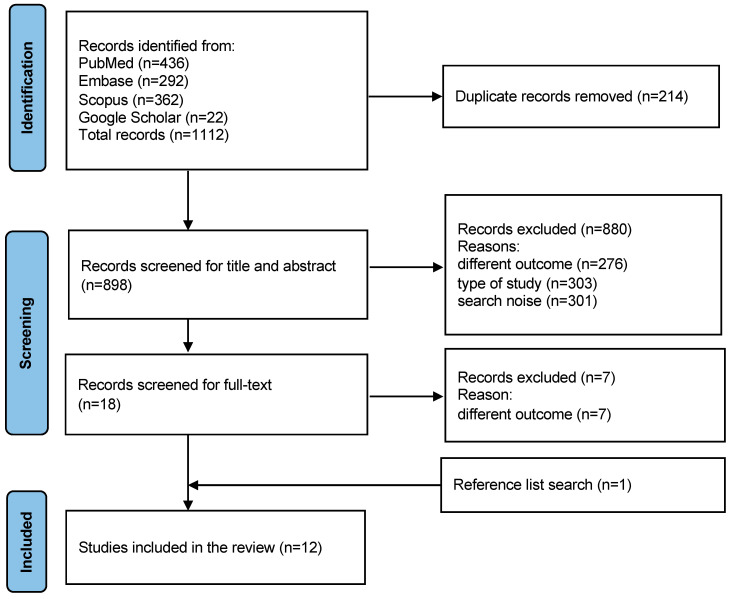
Prisma flow-chart.

A total of 1112 records were retrieved. After removing duplicates (n = 214), 899 papers were screened by title and abstract and 19 were selected. After full-text evaluation, 11 studies were selected and one additional article was found by searching in the studies’ reference lists, so 12 papers were included in this systematic review. The results of the search are displayed in the PRISMA flow-chart (Figure 1). The excluded studies after the full text evaluation are reported in the Supplementary File (Supplementary Materials File S3, Papers excluded after full-text evaluation). Cohen’s kappa value for inter-reviewer agreement was 0.57 at title and abstract screening (95.5% agreement) and 0.83 at full-text evaluation (96.1% agreement). 

### 3.2. Study Characteristics

All included studies were observational and published between 2008 and 2022. Regarding the samples, the number of infants considered varied from 20 to 155 [30,31], while for pregnant women, this number varied from 20 to 262 [31,32]. The maternal age ranged from 18 to 45 years [33,34]. All studies considered women without other clinical conditions than GDM. The type of delivery was declared in only six studies [30,31,32,35,36,37]. 

Regarding data on newborns (Table 1), the four studies that analyzed their microbiota found a depletion in *Firmicutes* and an enrichment in *Proteobacteria* [30,31,35,36]. All used oral swabs for microbiological analysis and considered data on birth weight and type of delivery. With regard to the data of women with/without GDM (Table 1 and Table 2), the dental evaluation of mothers was conducted in five studies [32,33,34,38,39], with only Yao et al. providing a comprehensive periodontal evaluation [34]. Oral microbiota detection was performed using oral swabs [30,31,35,36,40], in saliva [33,37,38], in plaque [32,33,34,38,39], and, in one study, both in plaque and saliva [33]. The microbial detection methods were sequencing [31,33,35,36,37,38,40,41], culturing [30,34], and PCR [32,39]. The results of the microbiological analysis showed that two studies did not find any statically significant difference in the oral microbiota of mothers with or without GDM [32,40], while an enrichment in anaerobic species was found in all the other studies [30,31,33,34,35,36,37,38,39,41], with a lower alpha diversity in mothers with GDM. A summary of the characteristics and results of the included studies is given in Table 1, Table 2 and Table 3.

### 3.3. Reporting Biases

Table 4a,b present the findings of the risk of bias assessment in the included studies. All the studies showed an overall high risk of bias [30,31,32,33,34,35,36,37,38,39,40,41].

### 3.4. Meta-Analysis

No quantitative synthesis was performed due to the high risk of bias found in the included studies.

### 3.5. Certainty of Evidence

The currently available literature does not provide evidence of an association between specific bacterial phyla or genera in the oral microbiota of infants and mothers and gestational diabetes. The high risk of bias could not allow for a quantitative analysis. Furthermore, the majority of research has not distinctly examined the microbial communities in individuals with periodontitis versus those with a healthy periodontium and its effect on infants. This is necessary to determine the specific differences attributed to infants’ oral microbiota in gestational diabetes mellitus (GDM) or periodontal conditions.

## 4. Discussion

Studies in the literature seem to indicate that the oral microbiota of infants of mothers with GDM varies from that of infants of mothers without GDM [30,31,35,36,40,42,43]; however, the present review does not confirm this hypothesis.

Dysbiosis, as an imbalance in the microbial community, cannot be transmitted directly from mother to offspring, but pathogens present in the imbalanced microbiota can be transmitted, adversely affecting the health of infants [6]. Hormonal changes during pregnancy lead to microbiota modifications and an increase in Gram-negative species; dysbiosis increases inflammation, which is enhanced by higher glucose levels [32]. When GDM occurs, anaerobic bacteria benefit from the higher glycemic values [20]. In addition, reactive oxygen and glycation species, inflammatory cytokines typical of GDM subjects, could negatively affect oral and systemic health [40].

The present review could not find evidence of a specific microbiological profile for the oral microbiota of infants and mothers with GDM, although an abundance of pathogenic species in infants and their mothers with GDM was reported [30,31,35,36,44]. Understanding how oral niches are colonized in the early period of life seems crucial for the early detection of microbiological conditions that may facilitate disease development. However, it should be emphasized that until the infant is edentulous, periodontal pathogens are transient in the oral cavity and the only possible colonization niche is the dorsum of the tongue [26]. As consequence, the need for well-conducted clinical trials in this field is high. However, to date, the lack of standardized methods for studying the oral microbiota has not given strength to the overall results of the studies. This led to the identification of bias in the measurement of outcomes and the reporting of results in almost all included articles. According to Nardi et al. [43], who analyzed many aspects of neonatal-maternal correlations of the oral microbiota, a possible alteration of the early oral microbiota of infants has been hypothesized not only if the child is born to a mother with GDM, but also in the case of maternal overweight, exposure to antibiotics during gestation, and in relation to the type of delivery and feeding. However, experimental protocols on maternal health and the oral microbiota of mothers and infants are needed to investigate this in depth and develop preventive strategies that can ensure a eubiotic oral microbiota in newborns. Most of the actual knowledge on neonatal microbiota from GDM mothers is about gut niches [45,46]. Modulation of neonatal gut microbiota is already a clinical practice in which the use of probiotics is a major determinant in the trajectory of its assembly [47,48,49]. For these reasons, as the oral cavity is the first part of the gastro-enteric tract, and, moreover, is the preferred route of administration of probiotics, its microbiota should be taken in great consideration when carrying out treatments on the gut microbiota.

Regarding the microbial profile of pregnant women, GDM led to an increase in anaerobic bacteria that are often associated with periodontal disease. However, the magnitude of how pre-pregnancy oral conditions could influence the outcomes is still unclear, as the studies did not check the subjects in a wide time range before and after pregnancy. This review showed an increase in Gram-negative and periodontal pathogens, although two studies could not find any difference. Previous research on women with GDM and periodontitis stage II, that focused specifically on *F. nucleatum* and *Capnocytophafaga* species, underlined that in cases of adverse pregnancy outcomes, these bacteria were found not only in dental plaque but also in cord blood and the peri-cervical vagina, to signify a possible blood transmission of these species [19]. Therefore, as many women with GDM develop type II diabetes, both women with prediabetic profiles and those with GDM should receive comprehensive health counselling, including weight management, healthy lifestyle behaviors (such as diet and physical activity), and dental counselling [50].

The composition of the newborn microbiota and species abundance are influenced by variables that may directly or indirectly perturb the microbial community during growth periods, but still, more evidence is needed to clear the modulation of oral microbiota when GDM occurs [51].

The limitations of this review are imputable to the reduced number of studies available, the high risk of bias detected, the low number of infants included in each sample, the different detection methods of the oral microbiota used, the different timing of sample collection, the lack of standardized dental evaluation for the mothers, and the different and sometimes contradictory results. Another significant limitation is that in only one study, women with cesarean deliveries were included to avoid microbial contamination from the vaginal tract [37]. Furthermore, it was impossible to gather data on prediabetic pregnant women and relative offspring in this review. Furthermore, it would be necessary to include data on subjects from different geographic areas to assess whether GDM and oral dysbiosis of the newborn vary according to other characteristics, such as maternal diet and different lifestyles [10,52].

Studying the origin and characteristics of newborn and maternal microbiota is essential for future tailored preventive/therapeutic actions; oral microbiota might be an optimal source of information due to the simplicity of sample collection. Thus, some authors suggest that oral swabs for pregnant women could be a more feasible detection method for GDM, as it does not imply blood collection nor the consumption of glucoside solutions [30,35].

Oral health is often underestimated by both clinicians and patients, even though periodontitis is one of the most common non-communicable diseases; indeed, in women with GDM, dental monitoring and, if necessary, related diagnostic and therapeutic procedures are required [53]. From the dental point of view, new research should evaluate the periodontal profile and analyze the inflammatory components that could result from the hyperglycemic condition and periodontal disease. It would be mandatory to investigate whether simple actions, such as regular professional oral hygiene in mothers with GDM, can improve the oral profile of infants in the short and long term.

## 5. Conclusions

Due to the important limitations of the included studies, there is no evidence that infants and their mothers with GDM have a distinctive oral microbiota. Furthermore, the effects of microbiological diversity found in some studies in children from mothers with GDM on general and oral health are not yet known. As the oral microbiota undergoes many changes, especially in the first two years of life, studies should monitor children for a long period to assess the role played by maternal GDM in the maturation of the ecological environment of the oral cavity of the child. Infants and their mothers with GDM should be regularly monitored by a multidisciplinary team, starting with the mother during pregnancy and continuing with mother and child in the first years of life. Regular check-ups are the best way to limit or manage periodontal disease in mothers and to motivate them to give the appropriate dental care to their infants, which may offset the possible transmission of potentially pathogenic bacteria due to GDM.

## Figures and Tables

**Table 1:** Characteristic of included studies focusing on microbiota of infants and mothers.

Study (Year)	He et al. (2019) [31]	Singh et al. (2020) [30]	Song et al. (2022) [35]	Wang et al. (2018) [36]
Country	China	China	China	China
Design	CO	CS	CO	CS
NB-GDM	9	75	20	11
NB-nGDM	11	80	34	9
M-GDM	9	75	20	77
M-nGDM	11	80	34	98
Natural birth	20	155	40	91
C-section	0	0	48	84
Weight NB-GDM (g)	2955.67 ± 296.56	3059.1	3200 ± 500	3410.31
Weight NB-nGDM (g)	3257.27 ± 291.38	3255.3	3200 ± 400	3386.16
Mean age M-GDM	28.44 ± 3.43	Nr	32.6 ± 4.3	Nr
Mean age M-nGDM	28.64 ± 3.17	Nr	30.5 ± 3.4	Nr
Outcome	Composition of NB microbiota	Composition of NB microbiota	Composition of NB microbiota	Composition of NB and M microbiota
M Dental evaluation	No	No	No	No
Type of sample	NB saliva oral swabs	NB saliva oral swabs	NB saliva oral swabs at 1 and 30 days from delivery	NB and M saliva oral swabs
Detection method	16S rRNA sequencing	Culturing	16S rRNA sequencing	16S rRNA sequencing
Bacteria depleted in N-GDM or M-GDM (phylum or genus)	NB Firmicutes	NB Firmicutes	Day 1NB Proteobacteria and Firmicutes; day 30 NB Firmicutes and Flavobacyeriales	M and NB Firmicutes. Leptotrichia
Bacteria enriched in N-GDM or M-GDM (phylum or genus)	NB Actinobacteria, Bacteroidetes, Proteobacteria, Tenericutes	NB Actinobacteria, Bacteroidetes, Proteobacteria, Tenericutes	Day 1 and 30 NB Proteobacteria, Actinobacteria, Bacteroidetes, Streptococcus	M and NB Proteobacteria and Lautropia
Results	Microbial diversity in NB-GDM was 3.48 ± 1.29 mean ± sd, in NB-nGDM 2.30 ± 0.97 (*p* < 0.05)	Microbial diversity in NB-GDM was 3.38 ± 1.21 mean ± sd, in NB-nGDM was 2.91 ± 0.91 (*p* = 0.02)	Microbial diversity at day 1 for NB-GDM was 3.36 ± 2.01 mean ± sd; for NB-nGDM, it was 4.0 ± 2.33 (*p* = 0.14). At day 30, it was 1.53 ± 1.07 for NB-GDM and 1.62 ± 0.68 for NB-nGDM (*p* = 0.34)	Bray–Curtis distance (calculated using the operational taxonomic unit abundance of the microbiome) was significantly smaller in NB-GDM than in NB-nGDM (*p* < 0.001)

CS: cross-sectional; CO: cohort; GDM: gestational diabetes mellitus; NB-GDM: infants from GDM mothers; NB-nGDM: infants from non-GDM mothers; M-GDM: mother with GDM; M-nGDM: mother, non-GDM; NB: infants; M: mothers/maternal; Nr: not reported; AUC: area under curve; sd: standard deviation.

**Table 2:** Characteristics of included studies focusing only on maternal microbiota.

Study (Year)	Dasanayake et al. (2008) [32]	Zhang et al. (2021) [33]	Yao et al. (2019) [34]	Ganiger et al. (2019) [39]	Li et al. (2020) [38]	Cortez et al. (2019) [40]	Xu et al. (2020) [37]	Crusell et al. (2020) [41]
Country	USA	China	China	India	China	Brazil	China	Denmark
Design	CS	CS	CS	CS	CS	CS	CS	CO
M-GDM	22	14	59	26	44	26	30	50
M-nGDM	240	55	59	26	67	42	31	161
Natural birth	184	Nr	Nr	Nr	Nr	Nr	0	Nr
C-section	64	Nr	Nr	Nr	Nr	Nr	61	Nr
Weight NB-GDM (g)	3039.0	Nr	Nr	Nr	Nr	Nr	3491 ± 384	Nr
Weight NB-nGDM (g)	3293.4	Nr	Nr	Nr	Nr	Nr	3440 ± 322	Nr
Age M-GDM	28.7	20–45	18–44	28.07 ± 3.75	31.5 ± 4.55	35.07	33.7 ± 4.7	34.4
Age M-nGDM	26.6	20–45	18–44	24.67 ± 3.69	30.41 ± 5.17	28.23	32.3 ± 4.3	33.3
Outcome	Microbiota composition and other periodontal parameters in M-GDB and M-nGDM with/without periodontitis	Microbiota composition in M-GDB and M-nGDM with/without periodontitis with/without GDM	Microbiota composition in M-GDB and M-nGDM (detection rate and number)	Periodontal status in M-GDM and M-nGDM	Microbiota data/composition from M-GDM and M-nGDM	Microbiota composition from M-GDM and M-nGDM	Microbiota data from M-GDM and M-nGDM	Microbiota composition from M-GDM and M-nGDM
Dental evaluation	Yes	Yes	Yes	Yes	Yes	No	No	No
Type of sample	Subgingival plaque	M saliva (1.5 mL) and supra- and subgingival plaque	Supra- and subgingival plaque	Subgingival plaque	M saliva and dental plaque	Oral swabs	M saliva sample (10 mL)	M saliva sample (2 mL)
Detection method	PCR	16S rRNA sequencing	Culturing	PCR	16S rRNA sequencing	16S rRNA sequencing	16S rRNA sequencing	16S rRNA sequencing
Bacteria depleted in M-GDM (phylum or genus)	No difference from M-nGDM	M-GDM with periodontitis: *Firmicutes*	M-GDM *Oral streptococci*, *Lactobacilli*	Nr	M-GDM saliva *Selenomonas Leptotrichia F16*; M-GDM plaque *Streptococcus*. *Veillonella*	No difference from M-nGDM	M-GDM *Bifidobacterium Leptotrichia*	M-GDM *Neisseria*, *Streptococcus*, *Actinobacillus paraheamolyticus*
Bacteria enriched in M-GDM (phylum or genus)	No difference from M-nGDM	M-GDM with periodontitis *Bacteroidetes Spirocheates Tenericutes Synergistes Porphyromonas Prevotella*	M-GDM *anaerobic bacteria*, *tubercolosis bacilli*, *actinomycetescapnocytophaga*	M-GDM *Porphyromonas Prevotella*	M-GDM saliva: *Lautropia*, *Neisseria*. *Neisseriales*; M-GDM plaque: *Lautropia*. *Neisseria*	No difference from M-nGDM	M-GDM *Neisseria*, *Porphyromonas*. *Prevotella*. *Streptococcus*. *Veillonella*	M-GDM *Prevotella Veillonella*, *Bacteroidales*, *Treponema*
**Results**	Periodontal parameters were not significantly different in the two groups (*p* = 0.38), both for *P. gingivalis* (*p* = 0.39) and *T. forsythia* (*p* = 0.73)	M-GDM with periodontitis had significant lower alpha diversity (*p* = 0.021) compared with M-nGDM with periodontitis	The number and detection rate of oral bacteria were higher in M-GDM than M-nGDM (Anaerobic bacteria *p* < 0.01)	*P. gingivalis* and *Prevotella intermedia* were higher in M-GDM than M-nGDM (*p* < 0.01 and *p* = 0.17, respectively)	Bray–Curtis intragroup distances of M-GDM group were significantly smaller than both the intragroup M-nGDM and intergroup M-GDM vs. M-nGDM distances (*p* < 0.001)	Microbiota did not show significant differences in phyla and genus among groups.	Alpha microbiota diversity was lower in M-GDM compared to M-nGDM (*p* = 0.04)	Number of observed OTUs decreased from pregnancy to postpartum (*p* < 0.01, *p* < 0.01) in both groups

S: cross-sectional; CO: cohort; GDM: gestational diabetes mellitus; NB-GDM: infants from GDM mothers; NB-nGDM: infants from non-GDM mothers; M-GDM: mother with GDM; M-nGDM: mother, non-GDM; NB: infants; M: mothers/maternal; Nr: not reported; AUC: area under curve; sd: standard deviation.

**Table 3:** Main characteristic and findings of included studies.

Topic	Oral Microbiota of Newborns from GDM Mothers	Oral Microbiota of GDM Mothers
Methods of microbiota detection	16r-RNA sequencing or culturing from oral swabs	16r-RNA or rDNA sequencing or PCR or culturing from oral swabs or saliva or plaque
Bacteria depleted	Mainly *Firmicutes*	Mainly *Firmicutes*, oral *Streptococci*, *Leptotrichia*
Bacteria enriched	Mainly *Actinobacteria*, *Bacteroidetes*, *Proteobacteria*	Mainly *Porphyromonas*, *Prevotella*, *Veillonella*
Statistical significance	All studies (4) showed significant differences between NB-GDM and NB-nGDM	Six studies of the eight included showed significant differences between M-GDM and M-nGDM.

GDM: gestational diabetes mellitus; NB-GDM: infants from GDM mothers; NB-nGDM: infants from non-GDM mothers; M-GDM: mother with GDM; M-nGDM: mother without GDM; PCR: polymerase chain reaction.

**Table 4:** Quality assessment of potential risk of bias using JBI Appraisal Checklist for cohort studies (a) and cross-sectional studies (b).

**Domain**	**Were the Two Groups Similar and Recruited from the Same Population?**	**Were the Exposures Measured Similarly to Assign People to Both Exposed and Unexposed Groups?**	**Was the Exposure Measured in a Valid and Reliable Way?**	**Were Confounding Factors Identified?**	**Were Strategies to Deal with Confounding Factors Stated?**	**Were the Groups/Participants Free of the Outcome at the Start of the Study (Or at the Moment of Exposure)?**	**Were the Outcomes Measured in a Valid and Reliable Way?**	**Was the Follow-Up Time Reported and Sufficient to Be Long Enough for Outcomes to Occur?**	**Was Follow-Up Complete, and If Not, Were the Reasons for Loss to Follow-Up Described and Explored?**	**Were Strategies to Address Incomplete Follow-Up Utilized?**	**Was Appropriate Statistical Analysis Used?**	**Overall Risk**
**Authors**												
Crusell et al. [41]												High
He et al. [31]												High
Song et al. [35]												High
(**a**)												
**Domain**		**Were the Criteria for Inclusion in the Sample Clearly Defined?**	**Were the Study Subjects and the Setting Described in Detail?**	**Was the Exposure Measured in a Valid and Reliable Way?**	**Were Objective, Standard Criteria Used for Measurement of the Condition?**	**Were Confounding Factors Identified?**	**Were Strategies to Deal with Confounding Factors Stated?**	**Were the Outcomes Measured in a Valid and Reliable Way?**	**Was Appropriate Statistical Analysis Used?**	**Overall Risk**		
**Authors**												
Cortez et al. [40]										High		
Dasanayake et al. [32]										High		
Ganiger et al. [39]										High		
Li et al. [38]										High	Legend	
Singh et al. [30]										High		
Wang et al. [36]										High	Yes	
Xu et al. [37]										High	Unclear	
Yao et al. [34]										High		
Zhang et al. [33]										High		
(**b**)												

## Supplementary Materials

The following supporting information can be downloaded at https://www.mdpi.com/article/10.3390/children11040421/s1: File S1: Search strategies; File S2: Papers excluded after full-text evalution; File S3: Prisma checklist
